# In(s) and out(s) of adolescent depression – Trajectories of development and recovery

**DOI:** 10.1016/j.bbih.2021.100382

**Published:** 2021-10-29

**Authors:** Zuzanna Zajkowska

**Affiliations:** Department of Psychological Medicine, Institute of Psychiatry, Psychology and Neuroscience, King's College London, UK

**Keywords:** Adolescent depression, Youth, Depression, Cortisol, HPA axis, Inflammation, Immune system, Neuroimaging, Brain-related abnormalities, Biomarkers, Psychological therapy, Psychotherapy

## Abstract

While the role of biological markers in understanding major depressive disorder (MDD) in adults have been studied extensively, less has been done to identify the biomarkers of MDD development and recovery in adolescence. With the majority of mental health disorders starting in adolescence, identifying biomarkers of transition and recovery from MDD early in life is critical for developing effective prevention strategies. Considering most of the child and adolescent populations come from low-and-middle-income countries (LMICs), it is vital to focus on adolescent populations in these settings. With most studies coming from high-income countries (HICs), evidence suggests that elevated morning cortisol levels including cortisol awakening response (CAR), increased peripheral inflammation and brain abnormalities such as cortico-limbic dysregulation or blunted activity in reward related regions in response to positive information are associated with MDD and being at-risk for MDD development in adolescence. We also find that some of the biological mechanisms of recovery from MDD, mainly normalisation in the cortico-limbic dysregulation, are reported following psychological therapy, suggesting shared pathways leading to MDD vulnerability and recovery. Although, only a few studies include adolescent populations. Understanding molecular mechanisms through which psychological interventions are effective, as well as molecular markers of transition to depression in individuals at-risk, are important to inform effective prevention and intervention strategies.

## Highlights

• Elevated cortisol and inflammation, and brain related abnormalities are linked with adolescent depression.
• Psychological therapy is linked with changes in the brain areas present in depression.
• Low number of studies in adolescent populations, with very few from low-and-middle income countries (LMICs).

## 1 Introduction

Major Depressive Disorder (MDD) is the third most frequent cause of death in adolescents globally and it will affect one in six people around the world during their lifetimes (Otte et al., 2016). However, surprisingly, little research has been done in understanding the biological mechanisms involved in the development of depression, as I recently reported in my systematic review of the worldwide literature looking at depression development in adolescence in the context of biological and environmental risk factors (Zajkowska et al., 2021).

Among the studies reviewed, only two were conducted in low-and-middle-income countries (LMICs), which is problematic considering that 90% of the world's adolescent population lives in LMICs (Kieling et al., 2011). The low-and-middle-income countries are defined as having gross national income per capita up to $12,695, which currently consists of 142 countries worldwide (World Bank Country and Le, 2021). Despite limited research in adolescent mental health in LMICs, there is sufficient evidence from the epidemiological studies to suggest the prevalence of mental health problems being present in up to 20% of adolescents in LMICs, which is comparable to adolescent populations from high-income-countries (HICs) (Kieling et al., 2011).

Most studies exploring the biological mechanisms of depression in adolescence focus on brain abnormalities, inflammation and hypothalamic-pituitary-adrenal (HPA) axis functioning (D'Acunto et al., 2019; Kennis et al., 2020; Colasanto et al., 2020; Miller et al., 2015). Findings from these studies to some extent support the results reported in adult depression (Jaworska et al., 2015; Dowlati et al., 2010a; Hepgul et al., 2016). For example, we observe that cortico-limbic dysregulation, increased peripheral inflammation and elevated cortisol levels are associated with subsequent depression onset (D'Acunto et al., 2019; Kennis et al., 2020; Miller et al., 2015; Lopez-Duran et al., 2009).

Interestingly, psychological therapy has been shown to normalise the cortico-limbic dysregulation present in depression in adults (Boccia et al., 2016). This suggests that biological alterations reported in depression might be reversible alongside the psychological improvement, and that this might happen via shared pathways leading to MDD vulnerability and recovery. Understanding these mechanisms is important if we were to plan effective prevention and intervention strategies.

Considering the high incidence of depression in the first decades of life, it is becoming clear that such interventions should be delivered as early as possible, to minimise the burden associated with this illness, and adolescence presents a window of opportunity to do that (Whiteford et al., 2013).

This review will focus on the biomarkers of depression development in adolescence and the evidence on the biomarkers of recovery from depression following psychological therapy - identifying the research gaps and informing future directions.

## 2 Hypothalamic-pituitary-adrenal (HPA) axis in depression

The dysregulation of the HPA axis functioning characterised by the elevated cortisol levels has been widely reported in adult depression. This is caused by the impaired negative feedback loop via the glucocorticoid receptor (GR) resistance, consequently resulting in inability to downregulate cortisol secretion (Nikkheslat et al., 2020). This leads to hypercortisolaemia which has been consistently reported in patients with depression who show elevated levels of morning cortisol - measured at one time point, elevated cortisol awakening response (CAR) – measured within the first hour of awakening, dysregulation in the diurnal cortisol secretion - measured at different time points throughout the day, and increased or blunted cortisol levels in response to stress – measured following experimental exposure to stressful task (Nikkheslat et al., 2020; Adam et al., 2010). Furthermore, experience of childhood traumatic events has been also associated with dysregulated cortisol levels in both adults and adolescents with depression (Nikkheslat et al., 2020; Harkness and Wynne-Edwards, 2011; Lu et al., 2016). For example, adults with history of childhood trauma showed higher CAR levels compared to those without such experience, regardless of the presence of depression (Lu et al., 2016). In another study, elevated diurnal cortisol was associated with severity of childhood trauma experiences in depressed patients with glucocorticoid resistance (Nikkheslat et al., 2020). In adolescents with mild depression, those who experienced childhood trauma showed increased levels of cortisol in response to Trier Social Stress Test (TSST) compared to adolescents without history of childhood trauma. Interestingly, the same study reported blunted cortisol levels in adolescents with severe depression regardless of early life adversity (Harkness and Wynne-Edwards, 2011). This may suggest that previously reported in adults blunted cortisol levels might be associated with severity and chronicity of depression also in adolescents (Burke et al., 2005). Of interest are also morning cortisol levels, especially cortisol awakening response (CAR), as that is when cortisol levels peak (around 40 ​min after awakening), in order to mobilise the body to start the day. Typically the increase in cortisol levels in CAR amount to 50–60% on top of waking levels of cortisol. Given that these are already elevated in depression, such increases are easy to depict when compared with healthy counterparts (Adam et al., 2010). Using CAR, as opposed to cortisol collected at one time point, has its advantage as it captures individual patterns of sleep-waking cycles. Several longitudinal studies from HICs suggest that elevated cortisol levels can predict depression onset in adolescence (Adam et al., 2010; Halligan et al., 2007). For example, elevated CAR predicted MDD development in adolescents a year and 2.5 years later (Adam et al., 2010, Vrshek-Schallhorn et al., 2013). Similarly, several longitudinal studies showed that adolescents who developed depression had higher morning cortisol levels measured prior to depression onset compared with healthy adolescents (Halligan et al., 2007; Owens et al., 2014; Goodyer et al., 2000, 2010). In addition to reported increase in morning cortisol levels, nocturnal urinary free cortisol (UFC) (Rao Constance and Poland, 2009) was found to be elevated in adolescents at high risk for MDD who subsequently developed depression compared with youth who did not develop depression.

Less clear is the link between morning cortisol levels and depression from the cross-sectional studies, where one study showed higher morning plasma cortisol in MDD compared with (healthy controls) HC (Birmaher et al., 1994), whereas another reported lower serum levels in MDD youth compared with HC (Dorn Ellen et al., 1996). Similarly, findings reporting how cortisol stress response relates to adolescent depression are equivocal. Two studies reported that adolescents with MDD had greater cortisol response to social stress tests compared with HC whilst the other two found no difference between the groups (Ming et al., 2017; Rao et al., 2008; Klimes-Dougan et al., 2014; Morris, 2014). Morris and colleagues used TSST to compare cortisol reactivity between adolescents with low and high risk for MDD and current MDD, and reported higher cortisol reactivity in the low-risk group compared with MDD and high-risk groups (Morris Chrystyna et al., 2017). Furthermore, adolescents with MDD had significantly higher levels of nocturnal UFC compared with HC (Rao and Poland, 2009). However, in another cross-sectional study which looked at 24 ​h UFC, no significant differences were found between MDD and HC groups (Dorn Ellen et al., 1996). Lastly, one study showed higher CAR levels in adolescents with MDD compared with healthy controls (Ulrike and Dirk, 2013).

### 2.1 HPA axis in depression - summary

Altogether, the evidence seems to suggest that elevated cortisol levels, in particular morning cortisol and CAR, precede depression onset in adolescence making them potential biomarkers of interest in predicting the development of this condition. However, findings from cross-sectional studies show mixed results. Altogether, studies in adolescents are only partly consistent with the findings reported in adults where HPA axis dysregulation is also reported in individuals with current depression (Lu et al., 2016). Considering that previous evidence in adults suggests that cortisol fluctuates with the severity of depression, where individuals with severe depression show blunted cortisol levels, those fluctuations might be starting in adolescence. As such, among individuals with current depression, there is a likelihood that some would experience severe depression and blunted cortisol response. Although limited in number, the findings reported in this review suggest that depression in adolescence is preceded by the elevated cortisol. One possible explanation of how the dysregulation might occur is through the experience of early life stress (Nikkheslat et al., 2020; Harkness and Wynne-Edwards, 2011; Cattaneo et al., 2015). This is important because it offers crucial information on where the prevention and intervention strategies should focus.

## 3 Inflammation in depression

Extensive research, mainly conducted in adult populations from high-income countries (HICs), suggests a role of inflammation and the innate immune system in the pathogenesis of depression, with several meta-analyses supporting these findings (Dowlati et al., 2010b; Baumeister et al., 2016; Enache et al., 2019). One of the mechanisms through which increased inflammation occurs links to hypercortisolaemia and GR resistance. Glucocorticoids have anti-inflammatory properties and while their receptors become resistant, the dowregulating mechanism fails which leads to increased inflammation (Miller and Raison, 2016). The “usual suspects” associated with depression tend to be elevated interleukins (ILs), interferons (IFNs) and acute phase proteins, including IL-1β, IL-6, IL-2, IFN-γ, tumour necrosis factor alpha (TNF-α) and C-reactive protein (CRP). Prospective studies suggest that increased inflammation precedes development of depression and therefore can be considered a risk factor for the development of this disorder (Hepgul et al., 2016). Studies in adolescents, for the most part, mirror the findings from the adult literature. Likewise, most of the studies come from HICs, with only one representing LMICs (Pallavi et al., 2015). A prospective study by Miller and colleagues reported that elevated blood IL-6 levels predicted development of MDD six-months later, however only in a group of adolescents who experienced childhood adversity. Furthermore, the same authors reported greater likelihood of having elevated blood CRP levels in adolescents who transitioned to develop MDD, but only in participants who experienced childhood adversity (Miller, 2012). In a large birth cohort study (ALSPAC), elevated IL-6 levels in childhood were associated with neurovegetative symptoms of depression, including sleep difficulties and fatigue, measured later in adolescence (Chu et al., 2019). Increased inflammation as a risk factor for depression in adolescents was also reported in the recent meta-analysis where increased CRP and IL-6 were associated with subsequent MDD development in children and adolescents (Colasanto et al., 2020).

Cross-sectional studies in adult depression suggest increased inflammatory markers being present in MDD compared with healthy individuals, and individuals who are responsive to treatment, as shown in studies from our, and other groups (Dowlati et al., 2010b; Cattaneo et al., 2020). However, cross-sectional studies in adolescent populations render mixed results. For example, three studies reported increased blood levels of IL-6 in adolescents with MDD compared with healthy controls (Pallavi et al., 2015; Gabbay et al., 2009). However, Byrne and colleagues did not find any difference in blood and salivary IL-6 levels between adolescents with MDD and healthy controls. Mixed results are reported for other cytokines too including TNF-α, which was reported higher in children and adolescents with MDD compared with healthy controls in one meta-analysis including four studies, however the difference reported did not reach statistical significance. Furthermore, some studies report no difference in TNF-α levels between the groups (Pallavi et al., 2015; Gabbay et al., 2009), or even lower levels associated with MDD (Byrne et al., 2013). Mixed findings are also found in other cytokines such as IL-2, where one study reported significantly increased blood IL-2 levels in adolescents with MDD compared with healthy controls (Pallavi et al., 2015), whereas another one showed no difference between the groups (Byrne et al., 2013). Similarly, one study reported higher blood levels of IFN-γ and IFN-γ/IL-4 ratio, with no difference in IL-4 levels alone, in adolescents with MDD compared with healthy participants (Gabbay et al., 2009), whilst two other studies showed no difference in IFN-γ between two groups (Pallavi et al., 2015), or even lower IFN-γ and IL-4 levels in MDD versus healthy controls (trend for significance) (Byrne et al., 2013). Lastly, two studies reported no difference in blood IL-1β between MDD and healthy controls (Pallavi et al., 2015; Gabbay et al., 2009), which is not consistent with the findings reported in adult depression.

### 3.1 Inflammation in depression - summary

Similarly to cortisol, more consistent findings from the longitudinal, and less so from cross-sectional studies, suggest that increased inflammation might precede depression development in adolescence, rather than being a marker of depression per se. As such, inflammation seems to play part in the trajectory of depression development in adolescence, and it might be accompanied by the experience of early life adversity as reported by Miller and colleagues (Miller, 2012). The lack of increased inflammation in current MDD, although inconsistent with the evidence in adults, can be attributed to age and chronicity of the illness, where elevated inflammation might become apparent as the state of depression persists longer, into adulthood (Dowlati et al., 2010b; Byrne et al., 2013). Furthermore, inconsistencies might arise from differences in the methodologies used across studies such as source of cytokine measurement, i.e., blood and saliva, or a sample source - economic and geographical settings. Although some studies suggest the comparability of the results measured in saliva and blood, cytokines concentrations can vary within the body and as such may not represent systemic inflammation (Byrne et al., 2013; La Fratta et al., 2018; Riis et al., 2014). Furthermore, all studies but one were from HICs (Pallavi et al., 2015). We simply do not have enough evidence to conclude whether biological changes associated with depression in adolescence are generalisable across different economic and geographical settings. Therefore, the methodological heterogeneity should be taken into account when interpreting the results.

## 4 Brain-related abnormalities in depression

Brain abnormalities including brain structure and activity have been reported in studies in adults and adolescents. Neuroimaging studies suggest fronto-limbic dysregulation with hyperactivity in limbic brain structures and hypoactivity in the prefrontal cortex being associated with depression and being at-risk for depression (Jaworska et al., 2015). For example, adolescents with MDD showed increased activity in limbic regions and reduced prefrontal cortex activity in response to stress compared with healthy adolescents (Ming et al., 2017; Klimes-Dougan et al., 2014). Furthermore, adolescents with MDD showed blunted activity in reward related regions in response to positive information across both, cross-sectional and longitudinal studies (Quevedo et al., 2017; Stringaris et al., 2015). Interestingly, such reduced activity in the right ventral striatum and left middle superior frontal gyrus at baseline predicted transition from healthy youth to youth with clinical symptoms of MDD, suggesting that it could be a potential biomarker of MDD development in adolescence (Stringaris et al., 2015). Neuroimaging studies in youth were also summarised in the meta-analysis which reported that children and adolescents with MDD showed hypoactivity in posterior insula during positive valence tasks and hyperactivity in dorsolateral prefrontal cortex superior temporal cortex during negative valence tasks, which suggested maladaptive emotional regulation during affective processing (Miller et al., 2015). Studies measuring white matter alterations using DTI technique reported that depressed adolescents showed lower fractional anisotropy (FA)-based connectivity centered on the right caudate, with connections to frontal gyri, insula, and anterior cingulate, compared with healthy controls (Tymofiyeva et al., 2017). However, no group differences were reported in the global network properties. LeWinn and colleagues (LeWinnColm et al., 2014), reported lower FA in networks related to emotion regulation including bilateral uncinate fasciculus, the limbic-cortical-striatal-thalamic circuit, corpus callosum, and anterior and superior corona radiata. In the longitudinal study lower FA values in the superior longitudinal fasciculi and the right cingulum-projecting to the hippocampus were associated with the subsequent MDD development (Huang and Rao, 2012).

Longitudinal findings from the EEG studies show sleep related abnormalities to be associated with subsequent MDD development in adolescents at-risk. These abnormalities include increased REM density and shorter latency, and lower temporal coherence (Rao Constance and Poland, 2009; VivekKutcher et al., 2002). Furthermore, frontal EEG asymmetry was investigated in a cross-sectional study where depressed adolescents showed lateral asymmetry with decreased left-sided frontal alpha activity, while no asymmetry was reported in HC (Grunewald EllenTrinkl et al., 2018).

### 4.1 Brain-related abnormalities in depression - summary

Overall, findings from the neuroimaging studies in depression in adolescence resonate with the findings reported in the adult literature including fronto-limbic dysregulation, blunted reward-related activity and white matter, and sleep related disruptions (Zajkowska et al., 2021). This suggests that neurobiological changes associated with the risk and development of depression are already apparent in adolescence. One theory explaining how this process occurs is diathesis stress model (Zajkowska et al., 2021). Chronic stress can lead to hyperactivation of the HPA axis via GR resistance, resulting in increased peripheral inflammation, which in turn can contribute to brain related abnormalities including structural and connectivity changes (Nikkheslat et al., 2020; Felger et al., 2016). Peripheral inflammation leads to increased inflammation in the brain which in turn can lead to functional changes in the neurocircuitry of the areas responsible for mood regulation and motivation such as neurotransmitter metabolism, synaptic plasticity, functional connectivity or neuroendocrine function (Felger et al., 2016; Capuron and Miller, 2011). It can also lead to decrease in neurogenesis leading to cognitive decline, commonly reported in depression (Marsland et al., 2008). As such, the framework of stress induced depression via the immune, neuroendocrine and brain changes is a good starting point in understanding the mechanisms underlying depression in adolescence, however we need more research to understand the exact mechanisms through which depression in adolescence develops and the role of wider context in this process, e.g., psychological and environmental settings.

## 5 Biomarkers of recovery from depression following psychological therapy

Psychological therapy, including cognitive behavioural therapy (CBT), group interpersonal therapy (IPT), group non-directive supportive therapy (NDST) and attachment-based family therapy are the first choice of treatment for adolescents with mild depression. If symptoms of depression persist, other forms of therapy are explored including brief psychosocial intervention (BPI), psychodynamic psychotherapy, family therapy and IPT for adolescents. The duration of each treatment would typically last up to 3 months with some variations across the type of therapy, e.g., CBT – 3 months, family therapy – minimum 15 sessions fortnightly, and psychodynamic psychotherapy – 30 sessions weekly. The choice of therapeutic modality depends on the individual's needs as determined by the clinician's assessment. Some therapeutic approaches focus on individual's reflective, cognitive, emotional and behavioural aspects in the therapeutic process, such as CBT; others might focus on more in-depth interpretation of the emotional states including exploration of past experiences, such as psychodynamic and psychoanalytical approaches; lastly, there are approaches that focus on relationships with others such as family therapy or IPT, or more directive approach such as BPI which includes psychoeducation and action oriented approach in tackling depression, i.e., guidance on building new skills and habits (Recommendations | Depress, 2021). However, very little research has been done to understand the molecular mechanisms of recovery from depression following psychological therapy in adolescence, where most evidence comes from the studies in adults.

For example, a pilot study by Gunlicks-Stoessel and colleagues showed that elevated levels of cortisol following a conflict task were linked with higher improvement in depressive symptoms following interpersonal therapy in adolescents (Gunlicks-Stoessel et al., 2013). However, a recent clinical trial reported that high evening cortisol levels were associated with slower decline in depressive symptoms in adolescents undergoing psychological therapy including CBT, short-term psychoanalytical psychotherapy (STPP) and BPI (Chadha et al., 2021). This is more in line with the studies in adults where, as reported in the meta-analysis by Fischer and colleagues, elevated HPA axis functioning in adults with depression predicted less favourable response to psychological treatment (Fischer et al., 2017).

There is also mixed evidence suggesting the effect of psychological therapy on inflammatory pathways with majority of the studies being conducted in adults. One meta-analysis, pooling evidence from 18 RCTs looking at the effect of different psychological interventions on a range of inflammatory markers, reported post-intervention decrease in CRP levels (O'Toole et al., 2018). However, another meta-analysis by Cristea and colleagues did not find any effect of psychological intervention on the immune markers. Similarly, they did not find any changes in cortisol outcome following psychological treatment (Cristea et al., 2019). A randomised clinical trial looking at the effect of cognitively-based compassion training (CBCT), an approach aiming at developing interpersonal skills leading to greater compassion and resilience, on CRP levels in adolescents in foster care who were exposed to early life adversity, showed that within the CBCT group the number of practice sessions correlated with the reduction in CRP levels from baseline to the end of treatment. However, no group differences were reported between CBCT and the waiting list group (Pace et al., 2013).

Neurobiological changes following psychological therapy for depression in adults have been reported in the literature nearly two decades ago. Goldapple and colleagues, using positron emission tomography (PET) technique, showed that treatment response following a course of cognitive behavioural therapy (CBT) was associated with metabolic increases in hippocampus and dorsal cingulate and decreases in dorsal and medial frontal cortex, areas implicated in the reward processing (Goldapple et al., 2004). Twelve years later, in the meta-analysis looking at neural underpinnings of psychotherapy in depression using functional magnetic resonance (fMRI) technique, Boccia and colleagues reported that psychotherapy led to changes in the activation of cortical networks, including temporal, frontal and cingulate areas, all of which are involved in cognitive processes including processing self-relevant information or memory encoding, retention and retrieval (Boccia et al., 2016; De Zubicaray et al., 2001). However, the most recent meta-analysis looking at randomised control trials (RCTs) of psychotherapy, yielded less conclusive results. It reported that certain type of psychotherapy (mind-body psychotherapy, which combines somatic and cognitive approaches) resulted in prefrontal asymmetry whereas no such effects were observed following CBT (Cristea et al., 2019). Of note, the number of RCTs reported in this meta-analysis was low and the heterogeneity between the studies was high. In adolescents, Straub and colleagues using fMRI and monetary incentive task found that reduction in depressive symptoms following five weeks of CBT was associated with changes across subgenual anterior cingulate cortex, amygdala and hippocampus, areas involved in emotion regulation and implicated in depression (Straub et al., 2015). In another study using electroencephalogram (EEG) method, adolescents who responded to CBT showed changes in the neural reactivity to reward, specifically lower reward positivity event-related potential predicted better therapeutic outcome (Burkhouse et al., 2016). Furthermore, increased functional connectivity in the reward-related circuitry which was reported in adolescents with depression, was reduced following CBT treatment. These findings suggest that CBT might act via the changes in the activity of the reward region in adolescents (Chattopadhyay et al., 2017).

### 5.1 Biomarkers of recovery from depression following psychological therapy - summary

Altogether, evidence from the studies in adults and adolescents suggests that psychological therapy, mainly based on the findings investigating CBT, can lead to changes in the neural functioning, in particular the reward-related circuitry which is implicated in depression (Goldapple et al., 2004; Straub et al., 2015; Burkhouse et al., 2016; Chattopadhyay et al., 2017). This review highlights the importance of exploring this area further, in particular conducting a systematic review or meta-analysis of the existing data would be a good way to gain a wider picture of the neural correlates associated with the recovery from depression in adolescents.

Less studies were performed looking at inflammation and cortisol in relation to the recovery from depression following psychological therapy, yielding mixed results. This could be explained by the high heterogeneity between the studies, i.e., methodology used, or type of therapy investigated. More studies are needed looking at specific types or specific components of therapy, e.g., behaviour activation or cognitive restructuring etc., to increase the accuracy of the results (Oud et al., 2019).

## 6 Discussion and future considerations

This review evaluated the existing evidence on biomarkers of MDD development and recovery in adolescence. The findings suggest that elevated peripheral inflammation and cortisol, as well as reward-related, white matter and sleep abnormalities are associated with depression in adolescence. In particular, there is a clear distinction between longitudinal and cross-sectional studies looking at immune markers and cortisol such that elevation in these biomarkers is more consistently reported as a factor preceding depression development rather than depression per se. As such, the predictive value of these biomarkers in the development of depression in adolescence might be of interest for the future investigations. Of note, these studies remain low in number and hardly any come from LMICs, making it difficult to generalise whether these findings stand true for adolescents globally. Even less has been done in terms of biological changes following psychological therapy in adolescents, with the exception to brain-related changes where there is some evidence suggesting that CBT affects neural circuitry, in particular in the areas of the brain related to reward processing, which seems to be consistent with the findings in adults. However, results from studies looking at inflammation and cortisol in association with depression recovery showed mixed results, and they were mostly conducted in adult populations. Considering differences in cognitive abilities to process information between adults and adolescents, we need more age specific studies that would capture biological processes associated with the outcomes of psychological therapy in adolescence (Miller et al., 2015; Braet et al., 2013). In line with diathesis-stress model, future research would also benefit from more longitudinal studies exploring the interaction between environmental stress factors and biological changes in the development of depression in adolescence (Zajkowska et al., 2021). Similarly, we need more longitudinal studies, exploring effectiveness of psychological therapy in adolescence and biological changes associated with it. Furthermore, an individual's development is influenced by the plethora of environmental factors including immediate social settings (family, peers etc.), wider culture settings, socio-economic status, exposure to violence, war conflict and many more (Zajkowska et al., 2021). As such, most findings reported in HICs might not generalisable to the global population of adolescents, of which 90% come from the LMICs, strongly suggesting that the future research should focus on those countries, especially considering that the magnitude and persistence of environmental stressors may affect the effectiveness of any psychological intervention in LMIC settings (ChiaraPapola et al., 2018). Furthermore, for this review we included the age range of 10–24, and the rationale for this is that adolescence is defined as a period of growth and transition from childhood to adulthood (Miller et al., 2015; outh and health ris, 2011; Sawyer et al., 2018). Such transitions, in particular changes in social roles and maturational changes in the prefrontal cortex, extend beyond 19 years of age (Sawyer et al., 2018; Miller et al., 2015). Therefore, it is important to capture the biological changes during this transition period across different cultures. However, this approach has its limitations as some biological and cognitive processes vary across different age groups within adolescence (10 to 19 years) and young adulthood (19 to 24 years) (Miller et al., 2015; Braet et al., 2013). Therefore, future studies should also consider focussing on specific age groups, e.g., early, middle and late adolescence and young adulthood, separately. Lastly, although the data presented in the first three sections of this article is partly based on the review of the literature performed for my previously published systematic review, which included global databases and no language restriction, the literature search for the remaining sections was restricted to articles in English language, which is a limitation that should be addressed in the future studies (Zajkowska et al., 2021).

## 7 Conclusions

**Fig. 1:**
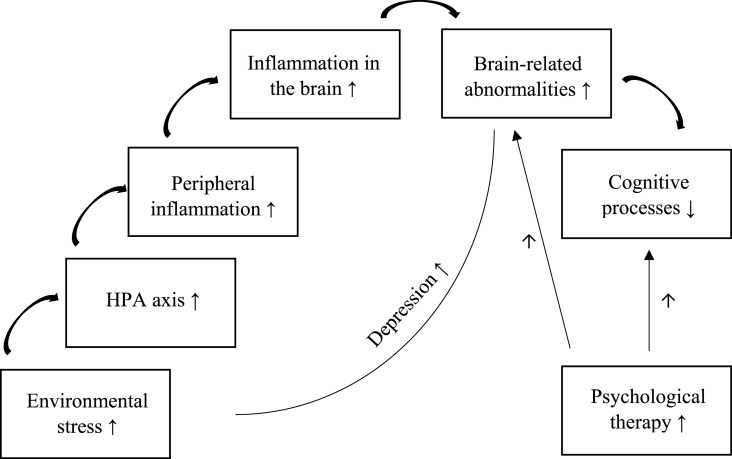
Schematic representation of the trajectory of depression development and recovery.

With the current lack of good prevention and treatment strategies for depression in adolescence, and an incomplete picture of what biological mechanisms lead to the development of this disorder, this short overview of the worldwide literature provides a good starting point. It highlights the research gap in understanding the biological mechanisms underlying depression development and recovery in adolescence. From the studies in adolescents, most of which originate from HICs, we find that the biological mechanisms of risk and presence of depression tend to support, to an extent, the findings reported in adult depression. However, biomarkers of recovery from depression in adolescence are less clear and under-studied. If we were to design effective prevention and intervention strategies, we need to understand the molecular mechanisms through which such interventions are effective, and whether they overlap with the biological pathways of transition to depression. Furthermore, we need to understand the biological changes as part of a wider context in which they occur including environmental and cognitive factors. As such, we need to conduct more research looking at the interplay between these factors and focussing on LMIC countries considering the vast majority of adolescent populations originate from these settings, and the generalisability of the findings from HICs cannot be assumed across other settings (Kieling et al., 2011). Given the high incidence of MDD in the first decades of life, it is very important that future research focuses on adolescent populations, including early, middle, and late adolescence and young adulthood, as that is the window of opportunity where we can effectively tackle the development of depression (Fig. 1).

## Declaration of competing interest

The author declares that there is no conflict of interests.
